# A Short Paper on Nervous Reflex Action

**Published:** 1880-12

**Authors:** Garrett Newkirk

**Affiliations:** Wenona


### A SHORT PAPER ON NERVOUS REFLEX ACTION.

By Garrett Newkirk, M. D., of Wenona.

*Mr. President and Gentlemen of the Illinois State Dental Society:*

I wish to call your attention—for a few minutes only of your valuable time—to a few thoughts concerning the importance of the phenomena belonging to what is known as reflex action.

It is certainly no less the duty of the dentist than the physician to make himself familiar with these phenomena, and, so far as possible, the principles involved in their production.

Since the discovery by Harvey of the circulation of the blood, there has been no one theory advanced in the domain of physiology so full of meaning and explanation; none so suggestive of unity on the one hand and numberless possibilities on the other.

As the circulation of the blood unites, in a sense, all the fluids and all the solids of the body, as the blood that is one moment passing through brain, may in the next be permeating the tissues of the kidney; so nervous action, properly understood, demonstrates the unity of the living force, and the sympathy and close relationship of parts exercising diverse functions.

Dim and indistinct ideas of an existing sympathy between parts remote from each other had long been entertained, but it was reserved for Dr. Marshall Hall to place the matter in clear light, in 1833, in his work on the "Structure and Functions of the Spinal Cord."

Professor Muller, of Berlin, almost simultaneously discovered the same facts, revealing to us the division of the spinal cord into motor and sensitive tracts, and the duplex character of the spinal nerves.

Lately in our own country, Dr. Brown Sequard has done more than any other to increase our knowledge of nervous action.

I can not, in the space to which I have determined to limit this paper, do more than glance at a few of the important features of the topic.

What do we understand by this term reflex action? The terms reflex and reflect, or reflected, are from the same root, with the same original meaning. We say a light is reflected, when it originates at a certain point, passes to a second, and from thence is sent in a different direction to a third. In this kind of reflection the second and third objects are but passive agents. They do not *act*, but are acted *upon*; they undergo practically no change. But in nervous reflexion, *all* the agents are active, *all undergo change*. Point first receives and conveys to point second a positive impression. There is a positive conversion of force at point second, and a positive effect at point third.

The point of departure in nervous reflection is the extremity of one or more sensitive nerve fibers. It may be one or ten thousand. The point of reflexion is a ganglion or mass of nerve matter in the spinal cord, the brain, or great sympathetic system.

The sensitive fiber or fibers receive an impression; force is transmitted to the central nervous mass, passes from thence by a motor root and nerve, or nerves, to the gland, muscle or other organ to be acted upon.

If I introduce something new in the order of arrangement I trust you will pardon me for employing a somewhat hackneyed illustration, that of comparing the nervous organization to a vast telegraph system. Let us call the brain the general depot, the head-quarters of the *general superintendent*. The ganglia of the spinal cord and great sympathetic we will say are head-quarters of the various division superintendents. The end of each nerve filament is a local station.

Then, let us imagine the stations everywhere so thick that a a pin-point can not find room between them, and we have a picture of a great empire within the human body, an empire teeming with life and energy, a vast system of organized forces, aggressive and resistive, with wonderful resources and unceasing industries, in which at every point the nerve sentinels keep watch and guard.

Carrying forward the illustration, let us notice, with regard to the nerve sentinels one important fact. Every local station has its own wire that goes direct and clear through to division head-quarters. The little wire—the little nerve—does not join a larger nerve and lose itself in it. No! Each little nerve filament keeps its individuality. It joins other filaments and travels side by side with them, in the same sheath, to the same central office, but it remains itself, ready to convey the message from the *local* office whence it starts.

We may speak of the great sciatic as one nerve, but in reality it is a bundle of ten thousand nerves, that are traveling together for mutual convenience and safety.

We speak of the great sensitive nerve of the face—the fifth pair with its three grand divisions—but it is many in one. Does the minute nerve, going from a single tooth-pulp to the main trunk, become really lost in it? Not by any means. We may not be able to trace it, but it undoubtedly goes clear through. It is the line of communication direct between the pulp and division head-quarters, where each special dispatch receives special recognition.

See how admirably adapted this is to local necessities. Suppose for instance the index finger receives an injury—a cut or burn. The nerve agents at that point telegraph immediately to the division superintendent at the cervical spine for repairs—an increased amount of blood and energy. The message goes straight through, on the index finger's special wire. It is transmitted further, to the conscious brain. There is no mistake about it. Both the brain and the spine know just where it comes from, know what the trouble is, and where to send supplies. But suppose the index finger's wire joined other wires, so as to lose its identity—all the wires from all the fingers and the thumb going up as one. How would the superintendent be able to locate the point of disturbance? How could he distinguish between these several members, know exactly all their needs, and so unfailingly supply them? According to that universal law of creation, by which *wherever a necessity exists*, the thing necessary is there present; each local station has its own wire, and the security of local interests is thus assured.

In order to attain a proper understanding of this subject, we must rid ourselves entirely of the notion that *small* nerves are branches simply of *big* nerves, shot off in various places like the boughs of a tree; or tributaries merely, like brooks and rivulets, whose waters meet, mingle and are lost in some larger channel. We must understand that each nerve, however small, is a *nerve by itself*; an independent citizen of the great vital commonwealth, responsible to head quarters for its conduct and good behavior; each filament attends to its own business, and reports to the division superintendent.

As previously stated, a *big* nerve so-called is a *big bundle of small nerves.*

Imagine ten thousand microscopic wires wrapped together in tissue paper, going up to the cord and brain. As they pass along, pinholes in the paper admit other wires, the sheath enlarges a little to make them room, until, as we approach the center, we find the little wires and the little bundles have made a great *cord*—just as fibres make threads, threads makes strands, and strands make a rope.

For simple illustration, I have taken the most simple form of reflex action—that of a nerve conveying an impression, as in the case of the index finger, and the nerve center responding at once with a message to the same part. This may be called direct reflexion, as when one's image is reflected to himself from a glass. We get the essential idea.

But as we analyze a drop of water and in a sense know the ocean, while we still fall infinitely short of a knowledge of its breadths and depths, so we may comprehend the simpler forms of nervous action while the vast and complicated movements of its waves and tides are wrapped in the mazes of the infinite. Comparatively we know but little about it, yet the little we do know is of great practical importance. Having learned our alphabet, we are beginning to spell and to read. From the simple we are reaching out after the more and more complex.

Allow me to present before your minds a picture in which is shown most beautifully both the theory and practical operation of nervous reflex power.

As during fetal life no air can enter the lungs, the muscles of respiration are not called into play, and that part of the nervous tract which is to exercise control of this function can not act. They are all ready. The wires are laid, the operators are in position at their respective instruments. The superintendent of respiration is in his place, *waiting for the first call*.

And now the time has come. The grand climax of maternal agony has been reached, and the man in minature ushered into the world.

But he does not breathe! The nerve operatives have not awakened from their long first sleep. Their sleep is deepened by the poisonous excess in the blood of carbonic gas.

There is no touch upon the keys of life. The division superintendent of the lungs hears no call.

The amount of oxygen in the blood grows less, and the skin is livid.

It is a moment of great anxiety. Will the child die? What can save it?

Nervous reflex action?

The physician understands something of its operation; he takes a handful of cold water and throws it violently upon the sensitive skin of the face and chest.

Ten thousand sleepers waken. Ten thousand wires thrill with messages to the nerve centers.

"What's that?" says the now aroused division superintendent. *A call for air?*

For the first time, but instantly, his fingers strike the keys. The diaphragm goes down, the costal muscles contract, the chest heaves; a vacuum is made, the oxygen comes in, the carbon passes out, the blood starts with a bound, and every fiber quivers with re-awakened life.

The vocal cords play their first little tune, beginning in a faint minor key, but swelling with each fresh inspiration into the first song of personal freedom.

The physician draws a sigh of relief, the paternal heart beats again; the exhausted mother finds strength to exclaim "Thank God!" then pillows her head to grateful sleep.

What an example of the conservation stored up in the possibilities of reflex action!

What should we think of the physician who would dare to remain in ignorance of all there is to be known of its practical benefits, and in his ignorance fail to strike the keys at the instant when a world of human happiness and destiny may depend upon a moment of time.

Examples might be indefinitely multiplied.

There is not a well-understood process going on anywhere in the body wherein reflex action may not be seen playing an important part.

Leaving it out as the key of mysteries, there is little we can properly understand.

By it parts remote from each other are brought near. They seem to understand one another's needs, and do practically, "bear one another's burdens."

By reflex action injured parts are restored, overworked and weary organs receive rest. They labor together in health and suffer together in disease.

When the head grows dizzy and faint, the stomach sickens, and the skin responds at every pore.

Food gives a normal irritation to the fauces, and we swallow. A feather gives an abnormal irritation, and we vomit.

The auditory nerve is irritated by the filing of a saw, or the performance of the amateur pianist, and our *teeth* are set on edge.

The grand offices of reflex action are the preservation of life and the conservation of force.

The nervous system presides over all, and every officer and man, from the general superintendent down to the most minute local agent, owes and cheerfully gives, *allegiance* to the *union, and stands by the constitution and the laws.*

Each citizen tries to perform his own duty faithfully, and subordinate to the public good. In the condition of general health each has sufficient to do, but not too much and everything moves smoothly.

But accident, sickness and disease work numerous exceptions to the rule of health.

Then it happens that nervous action becomes irregular. Abnormal irritation at one point is transmitted to another, in the form of pain or spasm. In such a case, *pain* being referred to a part *not* the seat of trouble, can not be relied on as a symptom standing alone.

Each local station having direct communication with cerebral or spinal head-quarters, the rule of health is that the superintendents at these points can tell with perfect accuracy where trouble exists and just what to do about it.

But suppose the superintendents are themselves afflicted, having become irritable from overwork and lack of rest; what marvel is it if they sometimes get confused, strike the wrong keys, and send wild messages?

By an investigation of these matters we arrive at more and more perfect conceptions of physiological unity.

We learn that when we put a needle-point upon the skin of the finger, we are not touching merely the skin, not merely the finger, but we are touching nerves, and *by the nerves, through the cord pricking* the *conscious brain.*

We are taught the important truth that we can not disintegrate this vast empire of the human body, take a fraction of it for treatment or experiment, and quietly ignore the great remainder. Each and every part is bound to the whole by ties that will not snap at our bidding.

We are taught that we must dip down deep into the myteries of *general* physiology and pathology, ere we may reasonably hope to deal rightly with the local and the special. Else how are we to read aright signs and symptoms, make correct diagnoses, or use proper means for the prevention and cure of diseased conditions?

Can any man expect to practice any specialty with satisfaction and security, with crude and indefinite ideas of general physiology, pathology and therapeutics, reflex nervous action and the philosophy of pain?

Has a man any guarantee against mistakes who does not understand that an indigestion, a uterine irritation, a dysmenorrhœa, a pregnancy, a rheumatic joint, an hysterical emotion, or a more or less distant local inflammation may be the loose key of the instrustrument causing unpleasant vibrations at the extremity of a dental nerve?

Not every physician understands as he ought how far disturbances elsewhere may be, by abnormal reflex action, referred to diseased and irritable tooth pulps, that an abnormal condition of the mouth and teeth is a most common cause of facial and cranial neuralgias, indigestion and non-assimulation of food; sometimes of deafness and disorders of vision, and frequently of nasal and bronchial catarrh.

Whatever we may say to the proposition that "Dentistry is properly a branch of Medicine and Surgery," the fact remains beyond dispute that we can not lay the foundations of our profession *too broad and deep* on the rock of general knowledge. The fact remains that we can not deal with any important organ of the body and that alone—touching one we touch all.

Many forces are playing on the many keys of life. It is an easy thing to produce discord, but if we are to make *harmony* while playing on *one key*, we must understand something of what the other keys are saying.

A case was under my observation of a very nervous gentleman who complained of neuralgic pain starting from a first bicuspid which had a sensitive cavity in it. The teeth next back of it were gone. The pain continued after filling. After two months of unsuccessful treatment, the pulp was destroyed and the root perfectly filled to the apex (as afterwards appeared when extracted). The trouble still continued, and the tooth was finally removed. In two or three weeks he returned with pain in the same location. Careful examination revealed a cavity with an exposed pulp in the wisdom tooth. This was extracted, and the pain was cured. In this case the bicuspid was lost for want of timely discovery of the real cause of the trouble. We *sometimes* find the trouble arising from a tooth in the opposite jaw and the other side of the mouth from the one designated as being the seat of the pain. The wisdom teeth are probably oftener the cause of these reflex effects than any others.

In one case pain in the larynx was caused by a diseased tooth. In another, a difficult dentition (of a wisdom tooth), caused blindness of one eye for twelve years, which was cured at once by the removal of the offending tooth. Spasm, threatening tetanus, is sometimes caused in the same way. In one case a diseased tooth caused insanity, which was cured by its removal.

Dr. Freeman had a case in which there was paralysis of the arm. This case was also relieved at once and completely by the removal of the offending tooth.

Dr. Hurtt: An abscess on the side of a molar root, the pulp in the tooth being still alive, produced partial paralysis of the lower lip and pain all along the side of the face. The destruction of the pulp relieved both the pain and the paralysis. Was it the irritation of the dental nerves, or the nerves of the gum tissue that caused the paralysis?

DR. J. A. DAVIS: In one case a diseased wisdom tooth was the cause of deafness, which was cured by the removal of the tooth. In another case a lower bicuspid was diseased and aching, and this and a sound upper one were removed, one after the other, without any relief, but the trouble was cured by the removal of a diseased wisdom tooth.

DR. PATTEN: A lady came to our town who had spent a great deal of money and taken a great deal of trouble for the treatment of sore eyes. The doctor brought her to me to see if there was anything the matter with her teeth. They appeared to be very good, but I was anxious to find *something* the matter with them, and after careful search succeeded in probing an exposed pulp through an obscure cavity in a wisdom tooth. The removal of this tooth cured the sore eyes.

In another case, a lady very hard of hearing came to have a lower wisdom tooth filled on the buccal side. I found the tooth very sensitive. Obtunding the sensitiveness by means of oil of cloves and morphine, was followed by immediate recovery of hearing.—*Trans. of the Illinois State Dental Society.*

